# Correction: Social determinants of vaccine hesitancy among the Lebanese parents: A cross-sectional Study

**DOI:** 10.1371/journal.pone.0353278

**Published:** 2026-07-06

**Authors:** Reem Awad, Mohamad Rahal, Anna Maria Henaine, Pascale Salameh, Robin Farah, Michele Cherfane, Reham Kotb, Diana Nakhoul, Reve Khaddaj, Nayla Habib, Katia Iskandar

The third and ninth authors’ names are spelled incorrectly. The correct names are: Anna Maria Henaine and Reve Khaddaj. The correct citation is: Awad R, Rahal M, Henaine AM, Salameh P, Farah R, Cherfane M, et al. (2026) Social determinants of vaccine hesitancy among the Lebanese parents: A cross-sectional Study. PLoS One 21(4): e0345153. https://doi.org/10.1371/journal.pone.0345153

The ORCID iD is missing for the ninth author. Please see the author’s ORCID iD here:

- Author Reve Khaddaj’s ORCID iD is: 0009-0000-9795-5767 (https://orcid.org/0009-0000-9795-5767).
