# Supplementary material for: Social determinants of vaccine hesitancy among the Lebanese parents: A cross-sectional Study
Source: PLoS One. 2026 Apr 1;21(4):e0345153. doi: 10.1371/journal.pone.0345153 (PMC13042773; doi:10.1371/journal.pone.0345153)
Supplement: Attachment — Submitted filename: Response to reviewers.docx [file pone.0345153.s001.docx]

The following changes are required before submission:

In the Methods section, please describe the vaccine hesitancy scale and include linear regression as the statistical procedure used to create Table 6. **Done**

Cite the references for each scale and provide a brief description. **Done**

Expand the description of the factor-analytic approach, specify the model, and cite the references. **Done**

Describe all derived variables and thresholds, explain how you chose the cut-points, and clarify the directionality.

In the results section, insert the tables near the corresponding texts. **Done**

Replace "their" by "my" in the sentence "I refuse to give the vaccine to their child", in the Methods. **Done**

State the purpose of VHL scale as done for the other ones.**Done**

The following changes are recommended:

Justify the reasons age group three or less was selected.

**This study focused on parents of children aged three years and below for several reasons: (1) This age group receives the majority of recommended childhood vaccines according to the CDC and Lebanese Ministry of Public Health vaccination schedules [11, 86], including critical vaccines such as DPT, MMR, polio, and hepatitis B; (2) Vaccine hesitancy decisions made during this period have the greatest impact on preventing vaccine-preventable diseases, as delayed or refused vaccinations during early childhood can lead to increased disease susceptibility and outbreaks**(1)**; (3) Previous research has shown that parental vaccine hesitancy is most pronounced during the first three years of life when vaccination schedules are most intensive [38]; and (4) Establishing positive vaccination behaviors during this critical period can influence parental attitudes toward future vaccinations throughout childhood and adolescence**(1)**.**

Describe how the parents were selected.

**Parents were recruited using snowball sampling, a non-probability sampling technique appropriate for hard-to-reach populations [36]. Inclusion criteria were: (1) Lebanese parents or legal guardians, (2) having at least one child aged three years or below, (3) residing in Lebanon, and (4) ability to read and understand either Arabic or English.**

Compare the results with similar or other countries.

**The findings of this study highlight the complex interplay of social determinants influencing childhood vaccine hesitancy among Lebanese parents. Socioeconomic status (SES), discrimination in medical settings, religious beliefs, and vaccine health literacy (VHL) were significant factors shaping parental attitudes toward vaccination. These findings align with global trends recognizing vaccine hesitancy as a multifaceted issue influenced by cultural, social, and economic contexts.**

**Key determinants of vaccine hesitancy**

**Socioeconomic Status and Discrimination: Lower SES was strongly associated with higher vaccine hesitancy, consistent with studies from other low- and middle-income countries (LMICs) where financial constraints and limited access to healthcare services exacerbate hesitancy [39]. Discrimination in medical settings further compounded this issue, with parents reporting higher discrimination more likely to distrust healthcare systems and delay or refuse vaccinations. Research examining health disparities demonstrated that even in settings where healthcare is provided free at the point of use, structural and institutional barriers within health systems significantly contribute to vaccine hesitancy**(2)**. Studies have documented systematically lower access to primary, inpatient, and outpatient care among populations experiencing healthcare inequities, coupled with higher likelihood of experiencing adverse encounters with healthcare systems, suggesting that healthcare access barriers and discriminatory practices rather than financial constraints alone drive hesitancy disparities**(2)**. This finding indicates the need for healthcare providers to address systemic biases through culturally sensitive communication and equitable care [40]. Healthcare authorities must actively involve marginalized communities in the development and implementation of healthcare initiatives to build trust and foster shared ownership, with participatory approaches proving effective in reducing perceptions of imposed interventions and promoting community buy-in**(2)**.**

**Religious Beliefs: Religious concerns, particularly regarding vaccine ingredients (e.g., animal-derived gelatin or non-halal components), played a significant role [41, 42]. While most respondents expressed neutrality or disagreement with these concerns, a notable minority cited religious beliefs as a barrier [43]. Tailored interventions, such as engaging religious leaders in vaccine advocacy and providing transparent information about vaccine ingredients, could help address these concerns, as demonstrated in other Muslim-majority countries [43].**

**Consistent with systematic reviews and meta-analysis , socioeconomic disparities and health literacy are universal determinants of vaccine hesitancy**(3)**. A global meta-analysis found parental vaccine hesitancy prevalence of 21.1% across over 30 countries, with lower-middle-income countries exhibiting rates ranging from 3.4% to 41.6%, reflecting substantial variability across geographic and socioeconomic contexts**(3,4)**. Unfavorable attitudes toward vaccination, including reduced perceptions of vaccine effectiveness and mistrust of health authorities, constitute major barriers to vaccine uptake across diverse settings [79] However, the prominence of discrimination and religious beliefs (Beta = 0.866, p < 0.001) appears more pronounced in Lebanon, necessitating locally-adapted public health strategies rather than Western imported approaches.**

**Vaccine Health Literacy: Higher VHL was associated with lower vaccine hesitancy, emphasizing the importance of health education in combating misinformation. Evidence from systematic reviews and multi-country studies confirms that individuals with high levels of vaccine literacy are significantly less likely to be unvaccinated, with this association remaining robust even after controlling for socioeconomic confounding factors**(5)**. The pathway through which vaccine literacy impacts hesitancy operates partially through mediating factors including confidence in vaccines, conspiracy beliefs, and complacency**(6)**. Parents with a health education background were significantly less hesitant, suggesting that targeted educational campaigns could improve vaccine acceptance[33]. Systematic reviews provide strong evidence that higher health literacy is inversely associated with vaccine hesitancy across diverse contexts, supporting public health approaches that prioritize increasing health literacy as a promising strategy for tackling vaccine hesitancy and reducing the burden of vaccine-preventable diseases**(5)**.**

**Healthcare Providers influence: Pediatricians were the most trusted source of vaccine information (43.7%) , similar to findings in Syria**(7) **. Cluster analysis reinforced that healthcare provider recommendations significantly impact vaccine decisions. The moderate influence of pharmacists suggests an untapped potential for expanding their role in vaccination programs. A systematic review demonstrated that pharmacist involvement consistently increased vaccine coverage** (8)**. In Lebanon, legal reforms enabling pharmacists to administer vaccines could improve access, particularly in underserved areas. Our findings also aligned with a Turkish study where higher education was associated with lower hesitancy, suggesting important contextual differences across Middle Eastern populations**(9)**.**

**Parents with health-related education background displayed significantly lower hesitancy, aligning with previous research showing that medical knowledge increases vaccines confidence**(10)**. Parents generally held positive attitudes toward all mandatory and recommended vaccines in Lebanon, consistent with a 2020-2021 national study** (11)**. These insights reinforce the need to enhance vaccine education, particularly among parents without a health background, and leverage trusted healthcare professionals to promote vaccine acceptance.**

**The potential impact of COVID-19-related distrust warrants consideration. Lebanon experienced inconsistent public health messaging and widespread misinformation circulating on social media platforms**(12)**. The pandemic period was also marked by the catastrophic August 2020 Beirut port explosion and Lebanon's worst economic crisis in modern history, creating a perfect storm of factors undermining public trust in institutions.**

**Previous research conducted in Lebanon found that negative past experiences with COVID-19 vaccination were significantly associated with reluctance to vaccinate children** (12)**. While our study did not directly assess COVID-19-specific attitudes or experiences, several findings suggest that pandemic-related trust erosion may have influenced our results: (1) the heightened discrimination scores may reflect negative COVID-19 vaccine rollout experiences,(2) a 19.8% reliance on social media as a primary information source is concerning given social media's role in amplifying misinformation, and (3) the association between lower SES and vaccine hesitancy may have been amplified by the pandemic's disproportionate economic impact on vulnerable populations.**

**The role of social media in Vaccine Decision-Making: Social media platforms significant sources of vaccine misinformation, with research demonstrating that non-scientific, sensational, or manipulative content on these platforms negatively affects public attitudes toward vaccines**(9)**. Social media misinformation are identified as a key factor influencing vaccine hesitancy in their systematic review of parents in Saudi Arabia, with vaccine hesitancy rates varying from 7.1% to 72.2%**(13)**. The mechanisms through which social media influences vaccine decisions are multifaceted: these platforms lack editorial oversight and frequently promote unverified, emotionally-charged claims about vaccine safety and side effects that have no scientific basis. Cagnotta (2025) explain that social media rapidly exposes users to both factual and emotional content, thus facilitating the spread of fear, doubt, and mistrust**(14)**. Misinformation regarding side effects, particularly when disseminated through digital and social media, can significantly distort individuals' perceptions**(9)**. Algorithmic amplification creates echo chambers where vaccine-skeptical beliefs are continuously reinforced, making users less likely to encounter evidence-based information. Misinformation and fake news shared on social media could influence parents' attitudes toward children's vaccination, with findings showing that female, more affluent, and more engaged social media users exhibited greater levels of vaccine hesitancy**(15)**. Research has demonstrated that misinformation spreads significantly faster than corrections, and visually compelling but misleading content, including emotional testimonials, anecdotes about alleged vaccine injuries, and conspiracy theories, tends to be more engaging and shareable than factual, science-based information. However, it is important to acknowledge that social media can also serve as a positive platform for disseminating accurate health information when properly leveraged. Evidence-based educational approaches are necessary to combat vaccine misinformation effectively. Cagnotta (2025) recommend a multi-pronged communication approach to address parental vaccine hesitancy: first, disseminating accessible health information using clear language and transparent data sources; second, deploying educational initiatives across schools, medical facilities, and digital platforms that encourage participation from communities and healthcare workers; third, organizing dialogue sessions that validate parental concerns while providing accurate information; and fourth, systematically identifying and refuting false claims through ongoing surveillance and fact-based responses**(14)**. Studies using rigorous trial methodologies have confirmed that educational programs significantly reduce vaccine reluctance among parents** (9)**. The manner in which vaccine information reaches parents proves critical for closing knowledge deficits and countering the swift dissemination of false narratives prevalent on social platforms. Communication strategies to enhance targeted communication include education through healthcare providers and dispelling social media misinformation, which are considered essential**(14)**. Without concerted counter-misinformation efforts, improvements in other determinants may have limited impact. Given Lebanon's high social media penetration (19.8% of parents in our study identified social media as their primary trusted source) and demonstrated influence on vaccine decisions, addressing social media-driven hesitancy should be a top priority. Educational strategies must be well-designed and evidence-based to effectively counter vaccine misinformation. Communication strategies to enhance targeted communication include education through healthcare providers and dispelling social media misinformation, which are considered essential. Without concerted counter-misinformation efforts, improvements in other determinants may have limited impact.**

Discuss the reliance of parents on social media for their medical information.

**Misinformation and fake news shared on social media could influence parents' attitudes toward children's vaccination, with findings showing that female, more affluent, and more engaged social media users exhibited greater levels of vaccine hesitancy. Research has demonstrated that misinformation spreads significantly faster than corrections, and visually compelling but misleading content, including emotional testimonials, anecdotes about alleged vaccine injuries, and conspiracy theories, tends to be more engaging and shareable than factual, science-based information. However, it is important to acknowledge that social media can also serve as a positive platform for disseminating accurate health information when properly leveraged. Evidence-based educational approaches are necessary to combat vaccine misinformation effectively. Cagnotta (2025) recommend a multi-pronged communication approach to address parental vaccine hesitancy: first, disseminating accessible health information using clear language and transparent data sources; second, deploying educational initiatives across schools, medical facilities, and digital platforms that encourage participation from communities and healthcare workers; third, organizing dialogue sessions that validate parental concerns while providing accurate information; and fourth, systematically identifying and refuting false claims through ongoing surveillance and fact-based responses. Studies using rigorous trial methodologies have confirmed that educational programs significantly reduce vaccine reluctance among parents . The manner in which vaccine information reaches parents proves critical for closing knowledge deficits and countering the swift dissemination of false narratives prevalent on social platforms. Communication strategies to enhance targeted communication include education through healthcare providers and dispelling social media misinformation, which are considered essential. Without concerted counter-misinformation efforts, improvements in other determinants may have limited impact.**

Report reliability statistics (e.g., Cronbach’s alpha) in the Results rather than in the Methods.

**Moved reliability and factor analysis to the resulsts section.**

Present Methods and Results in a stepwise manner aligned with measurement variables.

Change the order of Tables 1 and 2.

**Done**

Correct the following typos: In the Questionnaire, by the end of the paragraph, write parents' religious beliefs instead of believes; in the fourth sentence of Data Collection, modify as "A pilot-testing of the questionnaire included 20 parents from different gender, socio-economic and educational backgrounds"

**Done**

Results:

Provide the response rate.

**The response rate cannot be determined since the survey was sent online using the snowball technique.**

Modify the first line of second paragraph as ""Figure 1 shows that pediatricians are by far the most significant source of information"

**Done**

Five lines below, change neutral or disagreement to neutrality or disagreement.

**Done**

ANOVA results: write the first letter of polio, pneumococcal, and hepatitis in lower case.

**Done**

Table 5 description: define HCI.

**Done**

2. Please provide additional details regarding participant consent. In the ethics statement in the Methods and online submission information, please ensure that you have specified (1) whether consent was informed and (2) what type you obtained (for instance, written or verbal, and if verbal, how it was documented and witnessed). If your study included minors, state whether you obtained consent from parents or guardians. If the need for consent was waived by the ethics committee, please include this information.

If you are reporting a retrospective study of medical records or archived samples, please ensure that you have discussed whether all data were fully anonymized before you accessed them and/or whether the IRB or ethics committee waived the requirement for informed consent. If patients provided informed written consent to have data from their medical records used in research, please include this information.

**Done**

3. We note that your Data Availability Statement is currently as follows: All relevant data are within the manuscript and its Supporting Information files.

Please confirm at this time whether or not your submission contains all raw data required to replicate the results of your study. Authors must share the “minimal data set” for their submission. PLOS defines the minimal data set to consist of the data required to replicate all study findings reported in the article, as well as related metadata and methods (https://journals.plos.org/plosone/s/data-availability#loc-minimal-data-set-definition).

For example, authors should submit the following data:

- The values behind the means, standard deviations and other measures reported;
- The values used to build graphs;
- The points extracted from images for analysis.

Authors do not need to submit their entire data set if only a portion of the data was used in the reported study.

If your submission does not contain these data, please either upload them as Supporting Information files or deposit them to a stable, public repository and provide us with the relevant URLs, DOIs, or accession numbers. For a list of recommended repositories, please see https://journals.plos.org/plosone/s/recommended-repositories.

If there are ethical or legal restrictions on sharing a de-identified data set, please explain them in detail (e.g., data contain potentially sensitive information, data are owned by a third-party organization, etc.) and who has imposed them (e.g., an ethics committee). Please also provide contact information for a data access committee, ethics committee, or other institutional body to which data requests may be sent. If data are owned by a third party, please indicate how others may request data access.

**Data Availability Statement: The original data presented in the study are openly available through FigShare DOI: 10.6084/m9.figshare.28713395**

4. Please amend either the title on the online submission form (via Edit Submission) or the title in the manuscript so that they are identical.

**Done**

5. Please include your tables as part of your main manuscript and remove the individual files. Please note that supplementary tables (should remain/ be uploaded) as separate "supporting information" files

**Done**

6. If the reviewer comments include a recommendation to cite specific previously published works, please review and evaluate these publications to determine whether they are relevant and should be cited. There is no requirement to cite these works unless the editor has indicated otherwise.

Additional Editor Comments:

Reviewer #1:

1) Why did the authors choose the age group <=3 years of age? Why not broaden the age group?

This study focused on parents of children aged three years and below for several reasons: (1) This age group receives the majority of recommended childhood vaccines according to the CDC and Lebanese Ministry of Public Health vaccination schedules [11, 86], including critical vaccines such as DPT, MMR, polio, and hepatitis B; (2) Vaccine hesitancy decisions made during this period have the greatest impact on preventing vaccine-preventable diseases, as delayed or refused vaccinations during early childhood can lead to increased disease susceptibility and outbreaks; (3) Previous research has shown that parental vaccine hesitancy is most pronounced during the first three years of life when vaccination schedules are most intensive [38]; and (4) Establishing positive vaccination behaviors during this critical period can influence parental attitudes toward future vaccinations throughout childhood and adolescence.

2) How were the parents selected?
Parents were recruited using snowball sampling, a non-probability sampling technique appropriate for hard-to-reach populations [36]. Inclusion criteria were: (1) Lebanese parents or legal guardians, (2) having at least one child aged three years or below, (3) residing in Lebanon, and (4) ability to read and understand either Arabic or English.

The recruitment process began with an initial convenience sample of parents identified through personal networks. These initial participants were asked to share the survey link with other eligible parents in their networks via social media platforms (WhatsApp, Facebook, Instagram, LinkedIn). To maximize diversity, we specifically targeted parents from different governorates, socioeconomic backgrounds, and educational levels.

3) Discussion-how do these results compare to other countries and their beliefs? This is important to generalize the results to other countries.

4) Discussion-was there a factor of the distrust that developed during COVID influence some of these beliefs?

5) The results reveal that many parents are dependent on social media for their medical information-please expand on this as this is concerning and how can this be changed?

Reviewer #2: Minor revision

ABSTRACT
1) There was mentioned about involving questionnaire on 'political and economic instability’ but not mentioned in the Methods or any other sections of the main text.

**Deleted**

METHODS
1) in the sentence "I refuse to give the vaccine to their child", should the word "their" actually be replaced with "my"?

**Replaced**

2) Give the full term for SDOH as it is first mentioned in the main text, besides in the abstract.

**Done**

3) It was mentioned that the SES scale was used to capture respondent financial strains, DMS was to evaluate patients' self-reported experiences of discrimination in healthcare environments, DSES was used to examine parents' religious beliefs. But the purpose of VHL scale was not stated. Please provide at least a sentence that clearly mention the use of VHL scale.

**The Vaccine Health Literacy (VHL) Scale was used to assess parents' ability to obtain, understand, and use vaccine-related information to make informed vaccination decisions for their children. The scale consisted of 14 items divided into three subscales: functional literacy (understanding basic vaccine information), communicative literacy (ability to extract and apply vaccine information), and critical literacy (ability to critically analyze vaccine information). Responses were assessed using a 4-point Likert scale (1 = Never, 2 = Rarely, 3 = Sometimes, 4 = Often), with total scores ranging from 14 to 56. Higher scores indicated better vaccine health literacy. The scale demonstrated good internal consistency (Cronbach's alpha = 0.819) [33,34].**

4) It was stated that vaccine hesitancy scale was used as the dependent variable. But such scale was not described in the method. Please provide methods in determining the dependent variable. If the vaccine hesitancy scale was employed as claimed in the paper, clearly describe the scale like how other scales were described. Also, include explanation on the outcomes of vaccine hesitancy. Are the outcomes in binary outcomes since logistic regression was conducted? Or the outcomes are continuous, like the higher the score, the higher the hesitancy? If they are continuous, shouldn't Linear regression be performed rather than logistic regression?

**Indeed it is linear regression, not logistic regression.**

**The second section assessed vaccine hesitancy using a 23-item scale. Vaccine hesitancy was defined for participants as "a delay in acceptance or refusal of vaccines despite the availability of vaccine services." The scale measured two dimensions: vaccine hesitancy (16 items) and vaccine acceptance/trust (7 items). Respondents rated their level of agreement with statements concerning vaccination of children three years old and below using a 5-point Likert scale ranging from 1 (Strongly disagree) to 5 (Strongly agree). The vaccine hesitancy scale score was treated as a continuous variable (range: 23-115) in all analyses. Higher scores on the hesitancy subscale indicate greater vaccine hesitancy, while higher scores on the acceptance/trust subscale indicate more positive attitudes toward vaccination. The complete list of items and their factor loadings is presented in Table 1.**

RESULTS
1) What statistical test was used for results in Table 4? Perhaps can state in the table's footnote.

**Added it to footnote: Independent samples t-test for continuous variables; Chi-square test for categorical variables.**

2) Linear regression was used in Table 6, but the method was not mentioned in the Methods section, instead Logistic regression was mentioned.

**Indeed, corrected.**

3) Why other factors like sociodemographic factors were not included in the analysis of Linear regression in Table 6? Is there any specific reason?

**We considered only variable in the bivariate analysis with p< 0.05**

Reviewer #3: Reject

Methods
- Questionnaire provenance and content: Please clarify whether the questionnaire was newly developed or adapted from existing instruments. Validation Provide citations and brief descriptions for each scale (e.g., SES, DMS, VHL), including example items or domains, scoring, and interpretation. Define all abbreviations at first use.

**Done, thank you**

- Factor-analytic approach: Clearly describe the factor methods used, including whether the analysis was exploratory or confirmatory. Specify the factor model, provide (or reference) a path/construct diagram, and report key details (extraction and rotation methods, factor retention criteria, factor loadings, cross-loadings, and—if CFA—model fit indices such as CFI/TLI/RMSEA/SRMR).

**Reliability and validiy of the vaccine hesitancy scale**

**A factor analysis was conducted on responses from 251 participants to explore childhood vaccine hesitancy. The Kaiser–Meyer–Olkin measure indicated excellent sampling adequacy (KMO = 0.919), and Bartlett's test supported the factorability of the correlation matrix (χ² (253) = 6339.755, p < .001). Factor extraction was based on eigenvalues greater than 1 (Kaiser criterion). The analysis yielded two major components explaining 67.549% of the total variance. The first component (vaccine hesitancy) accounted for 37.948% of the variance (eigenvalue = 8.728), while the second component (vaccine acceptance/trust) explained an additional 29.600% (eigenvalue = 6.808).**

**Factor loadings ranged from 0.571 to 0.781 for Factor 1 and 0.803 to 0.882 for Factor 2, with no significant cross-loadings (all cross-loadings < 0.40). The correlation between the two components was relatively weak (r = 0.105), supporting the discriminant validity of the two factors. Reliability analysis yielded a Cronbach's alpha of 0.920, indicating excellent internal consistency. The scale demonstrated appropriate validity and reliability (Table 1).**

- Variable definitions and cut-points: Define all derived variables and thresholds (e.g., “high/low” discrimination scale, vaccine health literacy). Explain how cut-points were chosen (prior literature vs data-driven) and ensure directionality is clear so readers can interpret results consistently.

**Done**
Results

- Alignment of text with tables/figures: Ensure key claims in the text are supported by tables/figures. For example, the statement “Cluster 1 (those who refuse vaccines) exhibited the lowest uptake across all vaccines …” should be accompanied by a table or figure showing uptake by cluster with appropriate statistics.

**Done**

- Figure clarity (Figure 3): Improve the figure’s interpretability by enhancing labels Y axis. Also, I do not quit understand the interpretation of this figure.

**Figure 3 shows the influence of doctors, pharmacists, and social media have on vaccine hesitancy decisions. The legend in the figure mentions the level of infleunce either high, moderate or low. The three studied factors had moderate influence on vaccine hesitancy decisions. The doctors were the ones who had high influence on vaccine hesitancy decisions when compared to the other two in terms of high influence.**

Writing and organization
- Conciseness: The manuscript would benefit from substantial tightening for length and clarity.

**We shortened the Introduction and the discussion parts.**

- Logical flow: Present Methods and Results in a stepwise manner aligned with measurement variables.

**Done**

- Report reliability statistics (e.g., Cronbach’s alpha) in the Results rather than in the Methods.
I hope these comments are helpful in revising the manuscript.

**Done**
